# Understanding Race and Racism in Nursing: Insights from Aboriginal Nurses

**DOI:** 10.5402/2012/196437

**Published:** 2012-06-12

**Authors:** Adele Vukic, Charlotte Jesty, Sr. Veronica Mathews, Josephine Etowa

**Affiliations:** ^1^Dalhousie School of Nursing, Faculty of Health Professions, 1459 Oxford Street, Halifax, NS, Canada B3H 4R2; ^2^Mijua'ji'j Aqq Ni'n, Unama'ki Maternal Child Health, 201 Churchill Drive, Membertou, NS, Canada; ^3^Eskasoni Health Board, Eskasoni Community Health Center, Eskasoni, NS, Canada; ^4^School of Nursing, Faculty of Health Sciences, University of Ottawa, Ottawa, ON, Canada K1H 8M5

## Abstract

*Purpose*. Indigenous Peoples are underrepresented in the health professions. This paper examines indigenous identity and the quality and nature of nursing work-life. The knowledge generated should enhance strategies to increase representation of indigenous peoples in nursing to reduce health inequities. *Design*. Community-based participatory research employing Grounded Theory as the method was the design for this study. Theoretical sampling and constant comparison guided the data collection and analysis, and a number of validation strategies including member checks were employed to ensure rigor of the research process. *Sample*. Twenty-two Aboriginal nurses in Atlantic Canada. *Findings*. Six major themes emerged from the study: Cultural Context of Work-life, Becoming a Nurse, Navigating Nursing, Race Racism and Nursing, Socio-Political Context of Aboriginal Nursing, and Way Forward. Race and racism in nursing and related subthemes are the focus of this paper. *Implications*. The experiences of Aboriginal nurses as described in this paper illuminate the need to understand the interplay of race and racism in the health care system. Our paper concludes with Aboriginal nurses' suggestions for systemic change at various levels.

## 1. Introduction

Aboriginal people are significantly underrepresented in the health care professions. While statistics indicate that Aboriginal peoples comprise 3.8% of the Canadian population [1], currently less than 1% of Canadian nurses identify as having Aboriginal ancestry [2]. The relationship between Aboriginal Peoples and Western healthcare developed within a specific context of colonization and centralization in which medical professionals saw it as their role to help Aboriginal peoples “negotiate civilization,” while ignoring the detrimental effects of interference [3]. The history of colonization has had a significant impact on the health of Aboriginal peoples. The inclusion of Aboriginal health care professionals in decisions affecting their lives would lead to more equitable decision making in the factors that influence Aboriginal health. 

This paper presents the findings of a recent study that explored the work-life experiences of Aboriginal nurses in the Atlantic Provinces with the goal of strengthening current nursing recruitment and retention efforts. This paper was carried out with the recognition that efforts to diversify the nursing workforce must include an examination of the experiences of Aboriginal nurses already working within the system. Specifically, the research examined the nature of being an Aboriginal person in the nursing profession through the following research question: What are the work-life experiences of Aboriginal nurses working in health care systems in Atlantic Canada? Understanding Aboriginal nurses' work-life experiences informs how race, ethnicity, and culture intersect with nursing practice and education. 

## 2. Literature Review

A number of initiatives have been implemented over the past few years to enhance Aboriginal peoples' participation in the health professions. For instance, in response to the problem of nursing shortage in Canada, the Federal Government in 2004 allocated $100 M over 5 years to “create and implement strategies to increase the numbers of Aboriginal health professionals” [4]. Further, the Aboriginal Nurses Association of Canada (ANAC) has been involved in a number of initiatives. For example, ANAC met with nurses and stakeholders in May, 2006 to discuss ways of recruiting Aboriginal nurses, and equipping all nurses working with Aboriginal peoples with the tools necessary to provide culturally safe care [5]. ANAC [5] is currently partnering with a number of academic institutions to develop a mentoring program for Aboriginal nursing students. ANAC was formed in 1975 to “improve the health of Aboriginal people by supporting Aboriginal nurses and by promoting the development and practice of Aboriginal health nursing” ([6], page 34). According to ANAC, an increase in the number of practicing Aboriginal nurses is crucial to improving the health of Aboriginal communities overall [7]. The National Task Force on Recruitment and Retention Strategies [8] has identified the need for nursing leadership and support as a fundamental component of retaining practicing Aboriginal nurses in the profession. ANAC partnered with the Canadian Nursing Student Association (CNSA) in 2005 to cohost a strategic planning session designed to increase participation of Aboriginal nursing students in the CNSA and to develop a “framework supporting cultural diversity and safety in nursing education” ([9], page 10). 

 Several studies have examined the experiences of Aboriginal and minority nurses in Canada, Britain, and the United States ([10–20]). However, as Kulig and Grypma [21] note very little has been written about the history of Aboriginal nurses and despite their role in improving the health status of First Nations, Inuit, and Metis, very little research exists that explores the perspectives of Aboriginal nurses themselves. Kulig et al. [22] analyzed the results of a national survey of RNs working in rural and remote areas of Canada, in which 210 of the 3933 respondents self-identified as having Aboriginal or Metis ancestry. They found that 69.6% of Aboriginal nurses were originally from rural/remote communities, and that 66.7% chose to return to such areas because they wanted to work with their own people and raise families in smaller communities. 

Motivated by difficulties in recruiting and retaining Aboriginal students into nursing programs, Martin and Kipling [18] interviewed Aboriginal nursing students, Aboriginal nurses, and nursing faculty members to explore what kinds of factors shaped students' experiences. Participants identified inadequate funding and lack of available childcare as significant challenges, and they identified personal issues, lack of social support, racism, and conflicts with teachers as negative factors affecting their experiences in nursing education. These authors highlighted the exclusion of Aboriginal ways of knowing within the curricula and the lack of attentiveness to Aboriginal health care issues. They also advocated for collaboration among Aboriginal students and teachers in curriculum development and instructional design. Currently, there is limited research that examines the experiences of Aboriginal nurses already working in the health care field. An in-depth understanding of the work-life issues of Aboriginal nurses already working in the health care system is necessary for the development of future retention programs. The knowledge generated from our recent research on the work-life experiences of Aboriginal nurses in Atlantic Canada will inform the development of strategies to increase the representation of Aboriginal Peoples in nursing education, research, and practice. 

## 3. Methodology

This qualitative study was based on Grounded Theory as described by Glaser and Strauss [23] informed by a Community-Based Participatory Research (CBPR) approach and the principles of Ownership, Control, Access, and Possession (OCAP). In keeping with the tenets of (CBPR), and the OCAP principles, Aboriginal Atlantic Canadians were an integral part of the research as team members; as coprincipal investigator, community partners, and coinvestigators. The project also had a capacity-building component, which facilitated the hiring and mentoring of a research assistant of Aboriginal ancestry who participated in all aspects of the research. Ethical approval was granted by the University Research Ethics Board and Mi'kmaq Ethics Watch. Data collection consisted of unstructured qualitative interviews with Aboriginal registered nurses who were working (or had worked previously) in the various parts of Atlantic Canada. 

**Figure 1:**
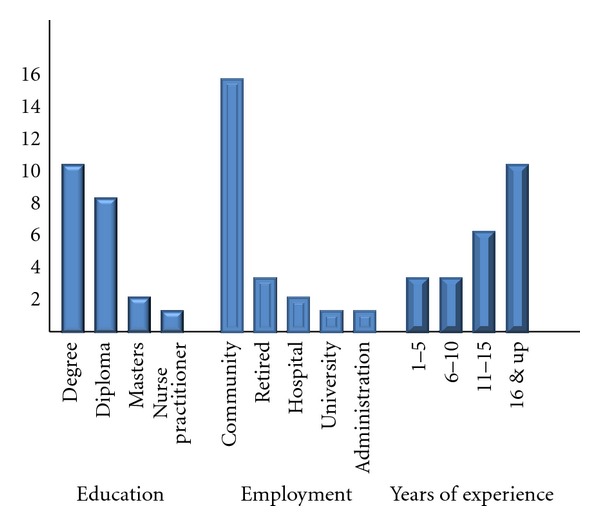
Atlantic Aboriginal nurses interviewed.

Participants were recruited through snowball sampling, an approach for selecting participants by asking well-situated people who to talk with and the list of interviewees increases as the research progresses [24]. Theoretical sampling technique guided the direction for further data collection and the achievement of data saturation. A total of 22 face to face interviews were conducted over a period of six months. Targeted effort was made to ensure maximum variation in the sample, thus study participants ranged from new graduates to retirees, young and more senior nurses who have held various nursing positions in the hospital, community, university; with education ranging from diploma, baccalaureate, nurse practitioner, and masters. See Figure 1 for details. All participants were female and the majority worked in a First Nations community. All interviews were conducted by one of the two academic members of the research team. The research assistant of Aboriginal ancestry accompanied some interviews to develop her Grounded Theory interviewing skills. In the interviews, participants were asked to respond to the following broad statement: “Tell me about your experience of being an Aboriginal nurse in the Atlantic Canadian health care system.” Most interviews were 60 to 90 minutes long with participants sharing many stories from why they went into nursing, their nursing education experience, their day to day lived experiences, and their personal career development. Interviews were transcribed verbatim. 

## 4. Data Analysis

Team members met to code and analyze the transcripts. Team members had data analysis meetings to code and analyze the information and Atlas-ti qualitative data software facilitated the management of this information. Data collection and analysis took place concurrently in keeping with the principles of Grounded Theory. Member-checks and peer-debriefing were used to evaluate the rigor of the research process and to enhance trustworthiness of the data. 

## 5. Findings

Six major themes emerged from the study: Cultural Context of Work-life, Becoming a Nurse, Navigating Nursing, Race Racism and Nursing, Socio-Political Context of Aboriginal Nursing, and Way Forward. Racism was a major theme revealed in the study findings and is described as “Race, Racism, and Nursing.” Participants talked about experiencing racism and of witnessing racism toward Aboriginal patients, and they shared some of the strategies they used to respond both to overt racism and to feelings of “otherness.” Participants did not blame individuals and consistently referred to the need for increased awareness of issues and institutional commitment to respond to racism. Some participants found that being Aboriginal was advantageous in that it enabled them to gain the trust of Aboriginal patients and to provide culturally safe care in hospital and community settings. The nurses in this study also talked about the burden of having to educate their colleagues about Aboriginal issues and of the underrepresentation of Aboriginal people in nursing, especially in leadership positions. The theme of racism will be described under the following subthemes; Experiencing Otherness, Responding to Discrimination, Tokenism versus Competence, and Cultural Safety and Diversity.

## 6. Experiencing Otherness 

The findings of this subtheme revealed a number of complex issues impacting on the work-life of Aboriginal nurses. Participants experienced personal discrimination from coworkers and patients, and several nurses interviewed also observed racism toward Aboriginal patients. One nurse asserted that racism toward Aboriginal patients in the hospital setting was more prominent than racism toward other minority patients,

*“I've seen blatant racism in the hospital setting that shouldn't be allowed and a lot of it is; and as an Aboriginal person I see more centered around Aboriginal more so than Black people or Asian people or anything like that. When they come in the hospital they're like “oh, they're probably drunk” or things like that.”*

This speaks to the lack of policies and resources to ensure that all nurses embrace cultural safety as an integral aspect of nursing care. Although another nurse felt angry and personally attacked when witnessing racism towards Aboriginal patients, both she and others feared that speaking up would put a strain on their relationships with coworkers. As one nurse remarked, “*I want to say more but I, I feel, I just don't know how to approach them without, without feeling like an outsider again*.” 

Though many participants identified experiences and observations of overt racism, some of the nurses expressed uncertainty around naming racism. As this statement explains, *“the comments can be very very subtle and you don't know whether you've just imagined a derogatory…tone*, *or if it was actually there*.*”* This uncertainty often prevented participants from speaking up when they thought they had observed racism, as they did not want to confront someone or make accusations if they were unsure about what they had witnessed. 

 The nurses in this study talked about fitting in, belonging, and being accepted. While some participants struggled with feeling as though they did not “fit in” with their non-Aboriginal coworkers or fellow students, others experienced friendship and acceptance. Some nurses learned with Aboriginal students in their nursing courses, which allowed them to find a sense of belonging within their own group:

*“I think because I had two other native students with me that we created our own sort of network of support. If I was by myself I might have had a different experience but I think having the other two nursing students together we could relate to each others' experiences yeah, and we could talk things out.”*

Others chose to enroll in nursing partly because they knew of Aboriginal students in these programs. As one nurse explained, “*the only reason I went there is because…I knew somebody…They were 2 years ahead of me…So I [knew] I wouldn't be alone.”*

Although some found acceptance with peers, others felt judged by instructors. One participant shared that instructors felt that she did not belong in higher education because she was Aboriginal, and that they even went so far as to tell her that *“You're not going to make a nurse, you'll never make a nurse, you're wasting your time.”* The following quote captures how some Aboriginal nurses, as students, recognized that instructors may never have had the experience of being on the other end of comments that can be interpreted as racist, and how some try to apologize.

*“And then when I walked back in the room, the instructor who had been in teaching the course tried to hug me in the, in the first thing, it was like one, you're invading my space, two just, just leave me be for a little while, just leave me be. And I think part of that was like not understanding the insult to begin with. And not being sensitive to, to having that experience.”*

In school, some felt that their personal learning style (the verbal learning and storytelling characteristic of Aboriginal culture) did not fit the norms of learning in the mainstream academic system and struggled with the written and concrete aspects of schoolwork. 

Participants felt judged by other nurses working in the hospital but found solace in caring for Aboriginal patients:

*“It was not a good experience. It wasn't like, I was alone. I felt I was alone, I was like, there was, especially around here, there's still racism eh? It was still here believe it or not. So when I worked in this hospital*…*there was only one person*…*one nurse that I got along with. I think she pitied me. But the other nurses I found, it was like, I don't know, I felt uncomfortable. I felt like they thought I didn't know anything. Or something like that. And my people were there, like the clients when they had native clients there like they were so glad to have a native nurse, like that made the difference….”*

Others found it rewarding to work in an Aboriginal community and to experience in turn a sense of belonging to a community.

*“Yes I worked seventy, eighty, ninety hours a week but I thrived on it. I, I, it made me feel whole to be able to help my community to help the people in my community. Part of that is the sense of belonging, the sense of being part of the community.” *

A few participants shared that they did not “fit in” to the mainstream nursing community partly due to the fact that English was their second language. Many pointed out that others sometimes misinterpreted their tone as bossy. Others found the transition from hospital nursing back to community nursing positive, in part due to language. As one nurse explained, *“here, I love it, I feel like I fit right in. I don't have to like hide the way I talk, like I'm not that good in English, like I kind of stutter a little and…they would say “You're not pronouncing that right.” I'd be like “I can't help it.”* Some participants confided that they would have felt more comfortable in nursing school as well as the hospital setting with a confidante who spoke Mi'kmaq.

The ability to speak a second language proved advantageous to the nurses who participated in this study. Participants commented how beneficial it was to be able to speak Mi'kmaq to Mi'kmaq patients. As one nurse explained, “*there's more of a bond between me and other Mi'kmaq patients…I can relate more to them.*” Aboriginal nurses who worked in a community setting provided examples of instances in which they used their own language to discuss with patients the need to seek particular treatments and pointed out that patients are often more likely to open up to them when they share the same language. 

## 7. Responding to Discrimination

This subtheme captured the ways in which the nurses responded to discrimination in their work as Aboriginal nurses. These include the use of both direct and indirect strategies, which varied depending on the individuals involved and on the particular situations they encountered. The strategies for responding to discrimination are categorized as; proving oneself, establishing credibility, educating others, passing as non Aboriginal and confronting the issue directly.

The nurses had to constantly prove themselves not only to the White community but also to their own people. In mainstream settings, establishing credibility meant proving one's competence. One nurse talked about being perceived as *that dumb girl*, and another talked about the need to prove oneself as follows:

*“We've had to prove to other people that we're just as smart as they are. The racism as I said in the very beginning, the racism is slowly changing but [province] is still, racism is alive and well. And it's to prove that I'm just as good as anybody else, if not better.” *Another echoed a similar sentiment, stating,* “I find myself having to prove my skills and myself to, to them Like the, my experiences, I feel when I get down to nitty gritty, at times I feel that I'm not respected because I'm Aboriginal.”*

As was the case for the nurses who worked in hospitals, the First Nations community nurses also had to prove their competence. As this comment explains:

*“Always proving, proving, proving to people that you can do it right. So yeah and I think that um in our communities like when I worked as a community health nurse, when I came across that kind of attitude again and it was there with my own people that I would challenge it. And I'd sometimes say well you know, I think you really need to rethink about that because you know we are trained in the very same way those nurses are trained and actually we probably can even be better because we understand a lot of the cultural, the language and what you're going through. And so oftentimes I think over time you just didn't need to say it anymore.” *

Yet for these nurses, establishing credibility also meant proving not only their competence, but also their loyalty to Aboriginal people and practices. As this quote explains:

*“Ok, so I am an Aboriginal nurse but I'm also a, you know, we also get educated in the mainstream so some of those ideas bringing into the community didn't go over quite as well. So when I first came either, well who does she think she is and yeah as if I was practicing as a mainstream nurse. Like you're supposed to be a native nurse why are you doing it that way.”*

These nurses had to prove that they would not abandon their communities, that they would remain loyal to their communities, and that they would remain true to Aboriginal culture and beliefs despite their mainstream schooling.

One of the ways in which participants responded to racism and discrimination was by educatingothers. Nurses spoke openly about personal experiences and observations of discrimination in order to educate many people about the fact that racism still occurs in hospitals and the need educate coworkers demonstrating racist behaviour by challenging stereotypes directly and by bringing to attention racial slurs. 

While some participants actively took on the role of educating coworkers, others felt it should not be their responsibility. For example “*I'm just not taking on the role of educating people about it anymore…Especially in the workplace because it's not my job. It's not my job*”. Although some nurses did address racist remarks, they did not believe that individual nurses should be responsible for providing this kind of education: “*It shouldn't be. It shouldn't be but I don't see who else is going to do it, right? I don't see my manager or patient care leader out there educating you know, the nurses. They probably don't even know.*” When confronting the issue of racism some participants reflected on the decision to speak up versus keep quiet in situations where one is confronting discrimination. Some participants felt comfortable addressing inappropriate language, but did not feel comfortable speaking up about broader issues. Some remained silent when overhearing discriminatory remarks in a hospital setting but defended personal racist attacks. 

Participants spoke at length about fear of educating coworkers. Others did not feel comfortable verbally confronting those who made inappropriate comments and therefore chose to remain silent. Some identified “speaking up” as a critical skill that could assist Aboriginal people in fighting discrimination:

*“I guess I haven't been taught the skills of conflict resolution or looking at a conflict. Um, maybe with more assertive skills or, or knowing how to address those things. I don't think we've ever been taught that. It's more if it happens it happens and you say nothing. Um, but maybe we need to start teaching people to speak up and say “I don't want to be spoken to that way” or “that's not how you should address me because I'm First Nations “you people”.” You know, those little things that maybe we need to educate one person at a time, I don't know but um, I really don't know what the answer is to that.”*

The issue of looking different versus passing as non-Aboriginal  arose in the interviews. Some participants looked different from their coworkers, while others had a more fair skin tone and eye color and could “pass” as another race. One nurse decided against working in the hospital because she was sure she would have been the only nurse with dark skin. Numerous participants commented on being able to pass as non-Aboriginal. Many felt that passing was beneficial in terms of their fitting in to the workplace. As this quote explains:

*“I think that was the first defense for me…See they didn't learn to know me as Mi'kmaq first, they learned to know me as a student nurse who is nice, would talk to you, give you a drink of water while you were you were you know, pleasant student. It helps when you don't have that barrier at first.”*

Many chose not to identify publicly as Aboriginal during training unless asked directly. One nurse explains how she chose to stay away from Aboriginal groups on campus for fear she would experience discrimination.

*“You know deep down I think that's why I never, because I didn't want anybody, you know…looking down on me because I was Aboriginal. As long as they didn't know I was Aboriginal, they treated me as equal. But I really felt that if they had known…once they know that, then it's different.”*

On several occasions, participants encountered patients who preferred a White nurse because they assumed that White nurses had more training. Some participants were quick to challenge these assumptions and to point out that they had equal training. Some felt that being an Aboriginal nurse provided a certain level of authority when visiting and/or participating in professional development with other communities. Some felt that their voice was heard by non-Aboriginals specifically because they were Aboriginal. 

Participants spoke of the high expectations communities had that they would be effective at *making the community better*. Many felt personal disappointment and the community's disappointment when positive changes did not occur immediately. Participants expressed that they felt very invested in their communities because they belonged to these communities in ways that extended beyond their nursing. 

## 8. Tokenism versus Competence

This theme refers to the complacent attitude associated with a marginal representation of Aboriginal people in a given health care organization. It is seen as racism because when an employer hires one Aboriginal person who happens to be successful he or she often thinks that the problem of discrimination has been successfully addressed. One of the nurses referred to this as the “Token Indian.” She went on to explain:

*“Somebody that, oh, we should have this minority, this minority, oh yes, we should have First Nations here too. And that's what I mean by token Indian, so that they can make sure that the representation is from our community.”*

Others chose to take advantage of this position as a “Token Indian” to seek out resources, programs, and services for the community. They also enjoyed the benefits of attending workshops and committees in order to connect to further resources. 

Similarly, another nurse experienced tokenism when she first applied for a job in a hospital. Her interview was very short and she was awarded the job immediately. She stated that she believed this was because “*they wanted a native nurse at the hospital, because all our First Nations people were off loaded to that local hospital. So I was hired on.*” Some participants addressed the topic in reference to being hired to work for a particular organization, As one nurse concluded, this led her to “toil” over whether she had been hired because she was Aboriginal, or because of her experience.

## 9. Cultural Safety and Diversity

Cultural Safety takes us beyond cultural awareness and acknowledgement of differences to addressing inequities witnessed because of differences [5]. Many participants spoke about the lack of understanding they witnessed among non-Aboriginal coworkers in both hospital and community settings regarding how to work effectively across ethno-cultural boundaries. One participant defined:

*“Cultural insensitivity to me, means that the nurses working in non Aboriginal clinic didn't really know too much about our culture and they were putting expectations on our people that they would expect from their own kind that were non Aboriginals. And I talked to them about it. I said, “You have to understand where these people are coming from.””*

Others commented on how unaccommodating hospitals are toward Aboriginal patients. These participants argued that hospitals needed to be more cooperative and flexible in respecting Aboriginal patients' culture, ideologies, and rituals. As an example, several participants addressed the practice exhibited by hospital personnel in restricting the number of visitors allowed at a patient's bedside at one time. One participant explains:

*“A Mi'kmaq would never let a Mi'kmaq die alone. But you know with non-native there maybe just 2 people by that person's side when they're dying. But if it's a Mi'kmaq, maybe you're going to see maybe 50 to 100 people you're going to see at the hospital, and they have to realize like in [city], one of my friend's father-in-law just passed away and then they are all praying like, they wanted to do like a really traditional like sacred ceremonial, they were not allowed. They all wanted to go help him, like when you pray in Mi'kmaq you can help them get to heaven faster, to get to the spirit world faster. They were there too long they were saying, so the nurses they called security, security came up and they told them to stop and told them to invite only 2 people at a time. So they didn't realize like about our culture.”*

Some participants asserted that allowances should be made for patients who wished to smoke a pipe to honor spirits. They concurred, not only should hospitals accommodate Aboriginal practices, but they should also attempt to understand the root of these practices: “*If an Aboriginal person wants to burn sweet grass how do you make those accommodations? Well if you don't understand why they're doing it then it's going to come, it's going to be a problem, you know?”* This nurse went on to say that many misunderstandings could be resolved if hospital staff were open to hearing and understanding where patients were coming from. 

The study findings revealed that some Aboriginal patients often feign understanding to save face when in fact they require clearer instructions for collecting specimens, preparing for medical procedures, and so forth. To facilitate better communication between Aboriginal patients and non-Aboriginal hospital staff, some Aboriginal nurses took on the unofficial role of cultural interpreters. Participants suggested that non-Aboriginal community nurses need to be more aware of the importance of relationships with patients. They also emphasized the necessity of home visits in ensuring one's effectiveness as a community nurse. 

The study finding also revealed the lack of formal diversity education in both education and workplace settings as a significant barrier to providing culturally safe care, some of the nurses recommended the integration of Aboriginal culture, customs, and ideologies in health professionals training curricula. Some nurses suggested that at times diversity education propagated generalizations and assumptions about Aboriginal culture. However, the nurses overall valued diversity in their education and some nurses took control of their own educatio because of this value. As this quote explains:

*“I wanted to make sure that I could incorporate Aboriginal health within my education as much as I could, because it wasn't a course in itself or the theme throughout the four years. So I kind of had to create those opportunities for myself.”*

Some participants observed a lack and in some cases absence of sufficient diversity education in the hospital workplace. As one participant described, “*And I know for like the non-Aboriginal nurse there's no cultural competency training or anything of the sort.*” Many participants provided suggestions on how to foster diversity in schools and workplace. Some recommended that Aboriginal nurses be invited into nursing classrooms to discuss cultural consciousness. As one participant commented “*I'd like to see that change. Maybe having guest speakers just who work in First Nations community nursing.*” Some advocated for an increase in the number of Aboriginal nursing teachers, while others also advocated for an increase in culturally safe instructors. Some asserted that a course focusing solely on Aboriginal health would benefit students. Others suggested that hospitals could incorporate cultural safety into their orientation processes, while others proposed that cultural safety be incorporated into “lunch and learn.” sessions. One participant asserted that non-Aboriginal and administrative hospital staff should be more informed about issues pertaining to diversity. As she points out:

*“ I now realize the complexity of culture and racism and that it is an ongoing battle as each new generation comes to the fore. Being an Aboriginal nurse, I have firsthand experience of living the “other” and therefore feel that I have a degree of sensitivity in this area. However, I can have the “other” as well when it comes to non-Aboriginal people. In working with non-Aboriginals in my community, I find that I am, at times, teaching the way of life and dispelling the generalizations perceived by non-Aboriginals.” *

All of the nurses interviewed in this study indicated that an increase in the number of Aboriginal Peoples in schools, in the nursing workforce, and in administrative positions would be welcomed and would benefit them for numerous reasons. They expressed the need for more Aboriginal Peoples on committees concerned with Aboriginal health. As one participant explained “*There was this big Aboriginal committee, not one Aboriginal name! Not one Aboriginal person on that committee! And that is so upsetting and discouraging.*” Other participants also mentioned that, although there were some nurse practitioners and Masters prepared Aboriginal nurses, they would like to see an increase in the number of Aboriginal nurses seeking higher education such as a nurse practitioner or master's level degrees. Some participants also commented on the need for an increase of Aboriginal nurses filling administrative and leadership role. One participant explained with regard to a particularly influential organization; “*There's nobody in a leadership position that's of First Nations, which is really I mean, it's wrong. People you know should be making decisions about their own lives like self-determination.*” Others talked about the advantages of working in a clinic with other Aboriginal nurses. For example: “*I think because we go way back from the beginning that we have this and we're from this community that we have this real good bond and real good, we work well together because we understand each other.”*

Some participants offered explanations for the shortage of Aboriginal nurses in the workforce such as the underrepresentation of Aboriginal nurses in tertiary care settings. They suggest this may be part of the reason why many Aboriginal nurses choose to work in Aboriginal communities where they work with other Aboriginal nursing colleagues and are more comfortable. Some nurses suggested that many young Aboriginal people were more likely to choose careers such as teaching and business over nursing because the significance of the work of nurse is often invisible. To address this, they suggested that high school students should be encouraged to take science classes from a young age so that they will be qualified for nursing education and to have more role models discussing Aboriginal nursing in the high schools. 

Other recommendations of the study include the use of innovative recruitment strategies such as facilitating summer camps in collaboration with Aboriginal communities, advertising in the field of Aboriginal nursing and promoting health fields through various venues, ensuring that Aboriginal nurses are represented at elementary and high school career days, and developing mentorship program for potential students who may be identified from career days or the summer camps. As one of the nurses asserted, Aboriginal nursing recruitment should focus on providing adequate support to students and on offering them a safe space in which to voice their concerns. She elaborated:

*“If we want more Aboriginal nurses*…*then we have to try and understand them, and maybe look at their culture, they're different, very different, and you need to understand that, and you've got to have some resources there to assist them in those times, that they're going to be, you know, because like I said they're moving away from home, they're adjusting to a different culture.”*

## 10. Discussion

Racism was a common thread in the stories of these nurses. The various manifestations of racism described by these nurses demonstrate the painful reality of everyday experiences of discrimination in nursing and the magnitude of this systemic issue. While some participants spoke of subtle racism, others spoke of blatant racism, and while some spoke of personal experiences of racism, others spoke of witnessing other Aboriginal people especially patients being discriminated against. These everyday incidents of discrimination are rooted in systemic structures that perpetuate differential and culturally unsafe treatment of Aboriginal people. Although racism is manifested in a number of ways including individual and/or systemic actions (or inaction in the face of need), it is more deeply rooted at the systemic level because the power to make decisions, to take collective action, and to allocate resources resides at this level [25]. Systemic racism is supported by policy action and inaction regarding persistently compromised health outcomes of people in positions of disadvantage [26]. Racism at the systemic level manifests in material conditions and in access to power, including differential access to health care services [27]. 

The many faces of racism highlighted in this paper reveal the insidious nature of the problem of race and racism in nursing. The nurses shared how being Aboriginal was important to them as they were able to inform coworkers about Aboriginal culture and advocated on behalf of Aboriginal patients in the hospital and the community. They also shared how being Aboriginal was advantageous and how they felt welcomed by Aboriginal patients in the hospitals and in Aboriginal communities. 

Participants offered innovative ideas for improving the system including specific suggestions for enhancing the recruitment and retention of Aboriginal nurses, such as summer camps, mentoring, role modeling, and increasing awareness of nursing as a career to high school students. An increase in the presence of Aboriginal nurses would create a greater support system and resources for Aboriginal nurses already working in hospitals and communities. The nurses in this study recommend that more Aboriginal nurses are needed in leadership positions to influence the policy and practices that affect Aboriginal Peoples. Currently only a few of the nurses in this study were in leadership positions. Senior leadership in the health care system should take some responsibility in ensuring the development and advancement of Aboriginal nurses into leadership positions and as Aboriginal nursing faculty. The time has come to support, recruit, and retain nurses in leadership positions so they are involved in influencing policy that affect Aboriginal people. Recruitment and retention is not simply a matter of more Aboriginal nurses to provide direct care.

While the majority of the Atlantic Aboriginal nurses in this study took it upon themselves to educate others in the context of their work environment they stressed the need for education on cultural safety with a concerted effort both in the nursing education and in hospitals. Nursing schools need to include cultural safety, conflict resolution, and negotiation skills in the curricula. Education of competent nurses capable of working effectively across ethno-racial boundaries should be everyone's problem. The study data highlights important insights into sensitive issues such as racism and discrimination, which are traditionally areas of discomfort. Nursing schools and workplace environments should have structures in place to address racism and promote diversity. These findings challenge nursing scholars to push the margins of nursing knowledge to explicate the complex social processes that influence human experience and consequently impact on health, especially the health of historically marginalized people. Some of their suggestions validate and reinforce current reports, research, and literature reviews on this topic. The literature supports the relevance of narrowing the gap between Aboriginal and non-Aboriginal health status by increasing involvement of Aboriginal Peoples in health delivery and policy-making [28, 29], and in recruitment of Aboriginal students into nursing programs and activities [9], as many of the participants in this study advocated.

A limitation to this study is that the majority of nurses participating in this study worked in the community as most of the Aboriginal nurses in the Atlantic Provinces are located in Aboriginal communities. A national study focusing on Aboriginal nurses in a variety of positions and in a variety of health care settings may provide further insights. Also, perspectives from non-Aboriginal nurses may constitute a broader analysis of race and racism in nursing. 

## 11. Conclusion

This study provides some direction for improving the quality of work-life of Aboriginal nurses to advance the health and health care of Aboriginal Peoples. In summary, this paper has provided some insight into the work-life experiences of Aboriginal nurses in Atlantic Canada, particularly with the understandings of race and racism in nursing, and how these have influenced their practice and decisions. Clearly, the nurses in this study have identified priorities for action to address the complex and systemic issues influencing the health and health services delivery for Aboriginal Peoples. The nurses in this study are eager to be involved in the health care system to affect change that promotes equity. As one nurse in this study concurs it is all about, “*Knowing my history, knowing what's been before me, knowing the wrongs that have happened to our community and wanting to make a change.”*
